# Research trends and hotspots on osteoporosis: a decade-long bibliometric and visualization analysis from 2014 to 2023

**DOI:** 10.3389/fmed.2024.1436486

**Published:** 2024-08-29

**Authors:** Song Zhang, Ye Liu, Weifeng Yu, Xiyao Gu

**Affiliations:** ^1^Department of Anesthesiology, Renji Hospital, Shanghai Jiao Tong University School of Medicine, Shanghai, China; ^2^Key Laboratory of Anesthesiology (Shanghai Jiao Tong University), Ministry of Education, Shanghai, China; ^3^Department of Anesthesiology, Yantai Affiliated Hospital of Binzhou Medical University, Yantai, China

**Keywords:** osteoporosis, bibliometric analysis, CiteSpace, VOSviewer, cited reference

## Abstract

**Background:**

Osteoporosis is characterized by diminished bone density and quality, compromised bone microstructure, and increased bone fragility, culminating in a heightened risk of fracture. Relatively few attempts have been made to survey the breadth of osteoporosis research using bibliometric approaches. This study aims to delineate the current landscape of osteoporosis research, offering clarity and visualization, while also identifying potential future directions for investigation.

**Methods:**

We retrieved and filtered articles and reviews pertaining to osteoporosis from the Web of Science Core Collection database, specifically the Science Citation Index Expanded (SCI-E) edition, spanning the years 2014 to 2023. Informatics tools such as CiteSpace and VOSviewer were employed to dissect the intellectual framework, discern trends, and pinpoint focal points of interest within osteoporosis research.

**Results:**

Our dataset comprised 33,928 osteoporosis-related publications, with a notable surge in annual publication numbers throughout the last decade. China and the United States lead in terms of research output. The University of California System contributed substantially to this body of work, with Amgen demonstrating the highest degree of centrality within the network. Cooper Cyrus emerged as a pivotal figure in the field. An analysis of highly-cited studies, co-citation networks, and keyword co-occurrence revealed that recent years have predominantly concentrated on elucidating mechanisms underlying osteoporosis, as well as its diagnosis, prevention, and treatment strategies. Burst detection analyses of citations and keywords highlighted osteoblasts, sarcopenia, gut microbiota, and denosumab as contemporary hotspots within osteoporosis research.

**Conclusion:**

This bibliometric analysis has provided a visual representation of the fundamental knowledge structure, prevailing trends, and key focal areas within osteoporosis research. The identification of osteoblasts, sarcopenia, gut microbiota, and denosumab as current hotspots may guide future research endeavors. Continued efforts directed at understanding the mechanisms, fracture outcomes, diagnostics, and therapeutics related to osteoporosis are anticipated to deepen our comprehension of this complex disease.

## 1 Introduction

Osteoporosis, characterized by diminished bone density and quality, compromised bone microstructure, and increased bone fragility leading to fracture, affects over 200 million people globally (1). Often referred to as “a silent epidemic,” its high incidence underscores its clinical significance (2, 3). Classified etiologically into primary and secondary types, primary osteoporosis typically manifests in individuals over 50 and is commonly linked with postmenopausal estrogen decline (4, 5), whereas secondary osteoporosis arises from underlying diseases or medications (6).

Fracture, the most frequent and severe complication of osteoporosis (7, 8), is a leading cause of disability, reduced mobility, loss of self-care ability, increased respiratory infections, bedsores, and imposes a substantial economic burden on families and society (9, 10). Osteoporosis often remains undiagnosed until a fracture occurs that necessitates surgery (8). With more than 9 million osteoporosis-related fractures reported annually worldwide (8), the lack of obvious symptoms frequently results in delayed prevention and treatment (7). The mechanisms behind osteoporosis and potential therapeutic targets remain elusive (11). Hence, both basic science and clinical research are crucial for advancing our understanding.

Bibliometric analysis offers a robust means for both quantitative and qualitative assessments of scholarly publications, enabling researchers to rapidly identify the frontiers and predict future trends within a given field (12–14). Tools such as CiteSpace and VOSviewer have been increasingly used in medical research to visualize information (15–18). Prior bibliometric studies have focused on specific aspects of osteoporosis, such as postmenopausal women (19), men (20), and rheumatoid arthritis patients (21). To date, no study has provided a bibliometric analysis of the entire osteoporosis field within the current decade. Using CiteSpace and VOSviewer, we conducted an exhaustive analysis of articles and reviews published between 2014 and 2023, aiming to elucidate the current state of osteoporosis research and uncover potential future directions for the field’s researchers.

## 2 Materials and methods

**Figure 1:**
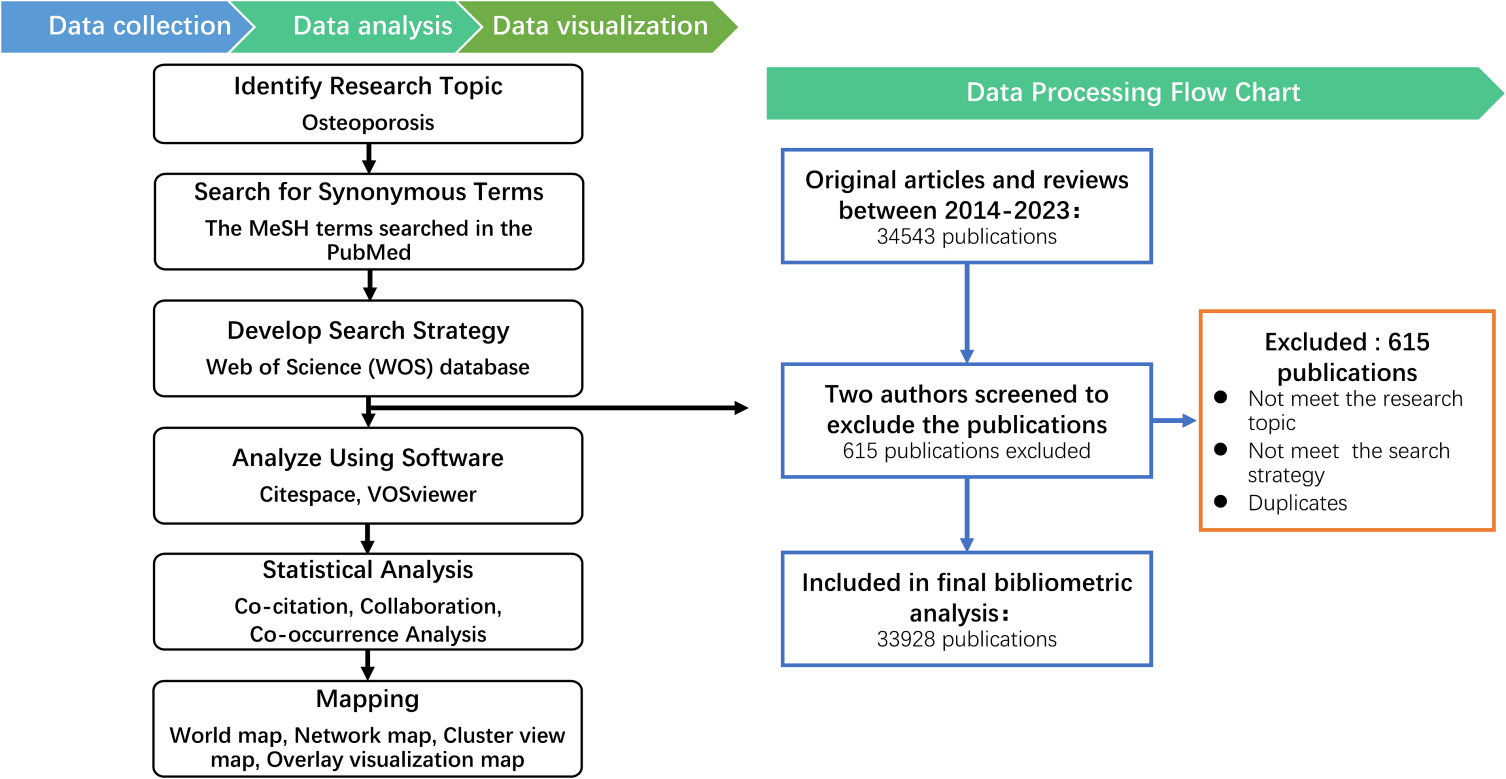
Bibliometrics processing of the information and suggested science mapping (Left) with Data processing flow chart for Osteoporosis (Right).

This original research follows the workflow outlined below: 1. Identify Research Topic: osteoporosis. 2. Search for Synonymous Terms: Using the PubMed database, we search for synonymous terms related to the research topic. 3. Develop Search Strategy: We establish a search strategy and retrieve relevant literature through the Web of Science (WOS) database. 4. Analyze Using Software: The retrieved data is analyzed using CiteSpace and VOSviewer. 5. Statistical Analysis: The results generated by the analysis software are statistically analyzed (Figure 1).

### 2.1 Data acquisition and search strategy

A comprehensive search for literature pertaining to osteoporosis was executed via the Web of Science Core Collection (WoSCC) database, specifically the Science Citation Index Expanded (SCI-E) edition. Our research terms are determined through the MeSH words related to osteoporosis in the PubMed database. The search strategy entailed the use of the following terms: [osteoporosis] OR [osteoporoses] OR [“bone loss, age-related”] OR [“age-related bone loss”] OR [“age-related bone losses”] OR [“bone loss, age related”] OR [“bone Losses, age-related”]. The timeframe for the search spanned from January 1, 2014, to December 31, 2023. The references type was limited to articles and reviews written in English language, with no constraints on data categories, for that articles and reviews contain a description of research and results (22, 23). All search operations and data downloads were conducted on a single day, March 16, 2024, to minimize discrepancies due to potential updates in the database. A 10-year period was applied to achieve the current hotspots. A total of 34,543 documents from 2014 to 2023 were found in the database. Two reviewers independently assessed and validated the retrieved publications relevant to the research topic. The exclusion criteria were: (1) Not meeting the research topic. (2) Not meeting the search strategy. (3) Duplicates. Finally, a total of 33,928 documents from 2014 to 2023, comprising 27,966 research articles and 5,962 reviews, were included in the study.

### 2.2 Data processing

The final dataset was exported in the format of “full record and cited references” for subsequent analysis. The bibliometric tools CiteSpace (version 6.2.R4, 64-bit, Drexel University, Philadelphia, PA, USA) and VOSviewer (version 1.6.18, Leiden University, Netherlands) were employed for further analytical purposes.

CiteSpace, a widely used tool for discerning the knowledge structure, distribution, and evolution within a field (24, 25), was utilized to visualize data pertaining to countries/regions, institutions, authors, as well as to identify clustered networks of co-cited references, references exhibiting the most significant citation bursts, and to detect keyword bursts. We began our analysis by setting appropriate parameters, including the time slice width and threshold. To simplify the network, we used the “Pathfinder Network Scaling Algorithm.” The “Logarithmization” option was applied to ensure a balanced link distribution. Additionally, we enabled the “burst citation detection” feature, which is specifically designed to identify emerging trends in scientific literature. The final presented keywords were obtained from further analysis through the abstracts, titles and official keywords from the references extracted in the study by Citespace.

VOSviewer, another bibliometric software developed by Professor van Eck and Waltman, possesses text mining functionalities that facilitate the extraction of critical parameters from an extensive corpus of scientific publications. VOSviewer clusters data by analyzing the frequency of exact keywords appearing in different documents. Accordingly, each node on the map represents an element, such as a country, institution, or keyword. The size of the nodes reflects the number of publications or the frequency of keywords or authors; the larger the node, the higher the number or frequency. The thickness of the lines connecting the nodes indicates the strength of co-occurrence or collaboration. The colors of the nodes and lines represent different clusters (22, 26).

The Online Analysis Platform of Literature Metrology^1^ is a web-based tool designed to examine yearly publication trends of the top 10 most productive countries/regions and explore collaborations among them. We first uploaded our dataset and selected “Country/Region” and “Year” as the dimensions for analysis. Using the “Collaboration Network Analysis” feature, we explored the collaboration relationships between different countries/regions. Additionally, we reviewed the results of the “Publication Trend Analysis” to understand the changes in the number of publications and citations in each country/region.

## 3 Results

### 3.1 Publication outputs

**Figure 2:**
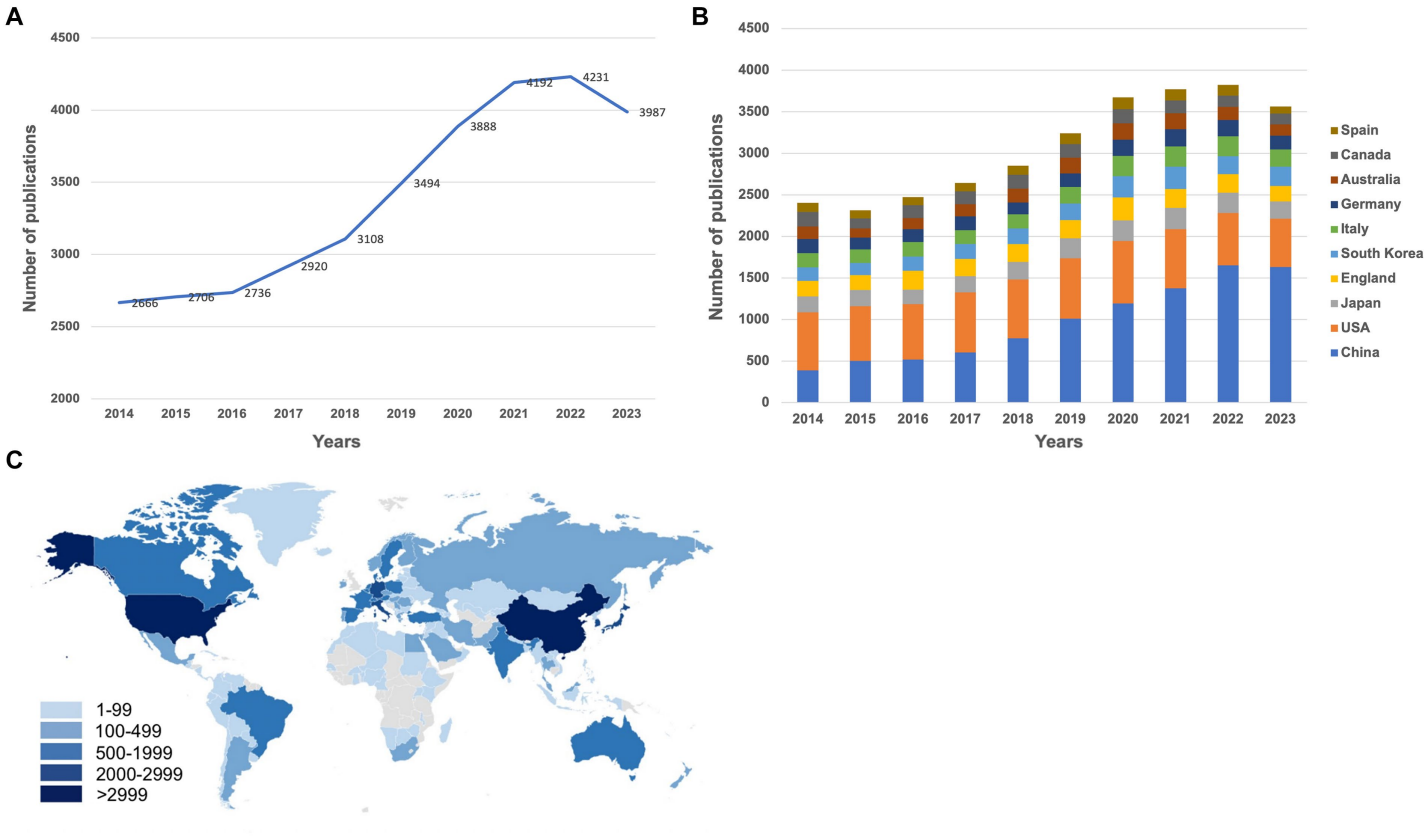
**(A)** The annual number of publications in osteoporosis research from 2014 to 2023. **(B)** The annual number of publications in the top 10 most productive countries from 2014 to 2023. **(C)** A world map depicting the contribution of each country/region based on publication counts. These figures were generated using the Online Analysis Platform of Literature Metrology (https://bibliometric.com/).

The count of publications serves as a direct indicator of the progression and evolution of scientific knowledge within a specific domain over time (21). A comprehensive total of 33,928 publications, comprising 27,966 research articles and 5,962 reviews, were retrieved from the Web of Science Core Collection (SCIE) database. With the exception of a minor reduction in the year 2023, there has been a consistent annual increment in the number of publications pertaining to osteoporosis, culminating in a maximum of 4,231 publications in the year 2022. It represents an approximate 1.58-fold increase compared to the count of 2,666 publications in 2014 (Figure 2A), thereby indicating a substantial growth in the scholarly interest dedicated to osteoporosis throughout the past decade.

### 3.2 Basic knowledge structures of osteoporosis

#### 3.2.1 Analysis of countries/regions

The top 10 contributing countries/regions to the field are China, the United States, Japan, England, South Korea, Italy, Germany, Australia, Canada, and Spain. China leads with the highest number of publications, totaling 9,657, followed by the United States with 6,866, both significantly outpacing other countries/regions (Table 1). The publishing volume from China has surged over the years, surpassing the United States in 2018 to take the lead, a position it has held ever since (Figure 2B). A world map, illustrated with a color gradient that signifies each country/region’s contribution, vividly shows that the bulk of the publications originate from North America, Western Europe, and East Asia (Figure 2C). These findings suggest that osteoporosis research is primarily concentrated within a select few countries/regions.

**Table 1:** Top 10 countries/region and institutions in terms of publications for osteoporosis .

Ranking	Country/Region	Publications	Institution	Publications
1	China	9,657	University of California system	818
2	USA	6,866	Harvard university	691
3	Japan	2,152	Shanghai Jiao Tong University	567
4	England	2,147	Institut National De La Sante et de la Recherche Medicale (inserm)	457
5	South Korea	2,015	Harvard Medical School	452
6	Italy	1,980	University of Oxford	423
7	Germany	1,703	University of Sheffield	422
8	Australia	1,569	University of London	418
9	Canada	1,528	University of Toronto	415
10	Spain	1,141	University of Southampton	393

**Figure 3:**
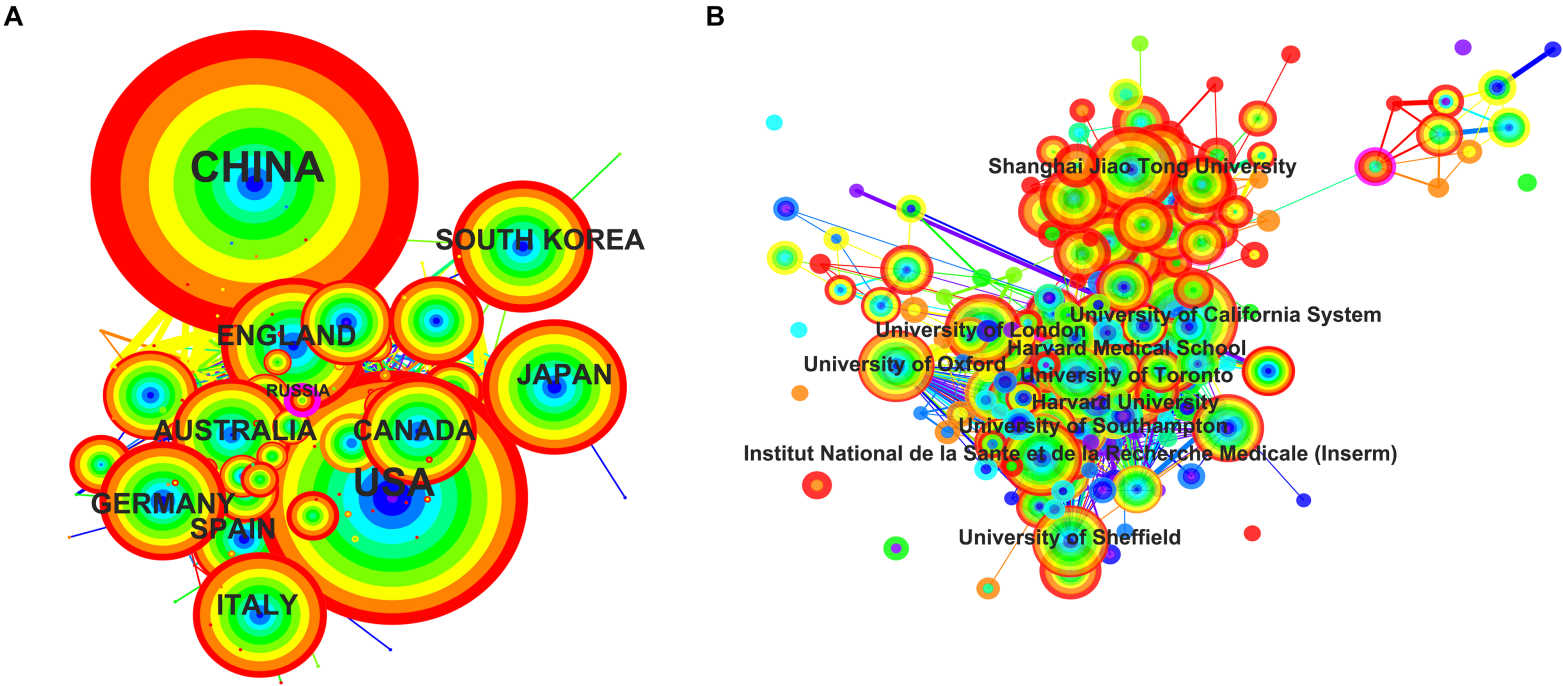
The network map of collaborating countries/regions **(A)** and institutions **(B)** in osteoporosis research. The collaboration map of countries/regions **(A)** and institutions **(B)** represents the collaborative relationships between countries/regions and institutions. The size of the circles indicates the number of published articles; the larger the diameter, the more articles published. The lines denote the collaboration strength. The color of the circles transitions from blue to red from the inside out, representing the publication years 2014–2023. These two figures are generated using the Citespace software.

The examination of country/regional cooperation elucidates the collaborative engagements between a particular country/region and others within a specified research area (27). By segmenting years into individual increments and applying a threshold that selects the top 100 contributors, we obtained data pertaining to the most prolific 100 countries/regions in terms of annual publications. Upon generating the countries/regions cooperation network map with CiteSpace, it identified 142 nodes interlinked by 1,430 connections, signifying that these 142 countries/regions have engaged in 1430 cooperative ventures (Figure 3A). Additionally, the density of 0.1428 for the national cooperation map suggests that inter-country/regional collaborations are infrequent. Furthermore, centrality in the bibliometric analysis serves as an indicator for the intensity of these partnerships, describe network characteristics, reflecting the influence and importance of nodes within the network, where nodes exceeding a centrality value of 0.1 indicate significant influence (27). Russia is the sole entity with a centrality measure reaching 0.1. These findings collectively underscore that international cooperation is fragmentary and requires augmentation.

#### 3.2.2 Analysis of institutions

Over 18,000 institutions have been involved in osteoporosis research, with more than 1,000 of them contributing over 20 publications each, highlighting the field’s widespread interest. Table 1 presents the top 10 most productive institutions (Table 1, also see in Supplemental Table 1 for top 100). Notably, the University of California System leads with a total contribution of 818 publications, followed by Harvard University with 697 publications, and Shanghai Jiao Tong University with 567. By segmenting the years into individual units and applying a selection criterion of top 100 contributors, data pertaining to the 100 most active institutions annually were extracted. The resulting institutions’ cooperation network map consisted of 234 nodes interlinked by 1,930 connections, with a density of 0.0708, suggesting a decentralized research distribution amongst these institutions (Figure 3B). Institutions such as Amgen (0.17), Naval Medical University (0.16), University of Manitoba (0.13), National Yang Ming Chiao Tung University (0.13), Chinese University of Hong Kong (0.12), and University of Sheffield (0.1) had centrality measures equal to or exceeding 0.1. This indicates that there is room for further enhancement of inter-institutional collaborations.

#### 3.2.3 Analysis of authors

**Figure 4:**
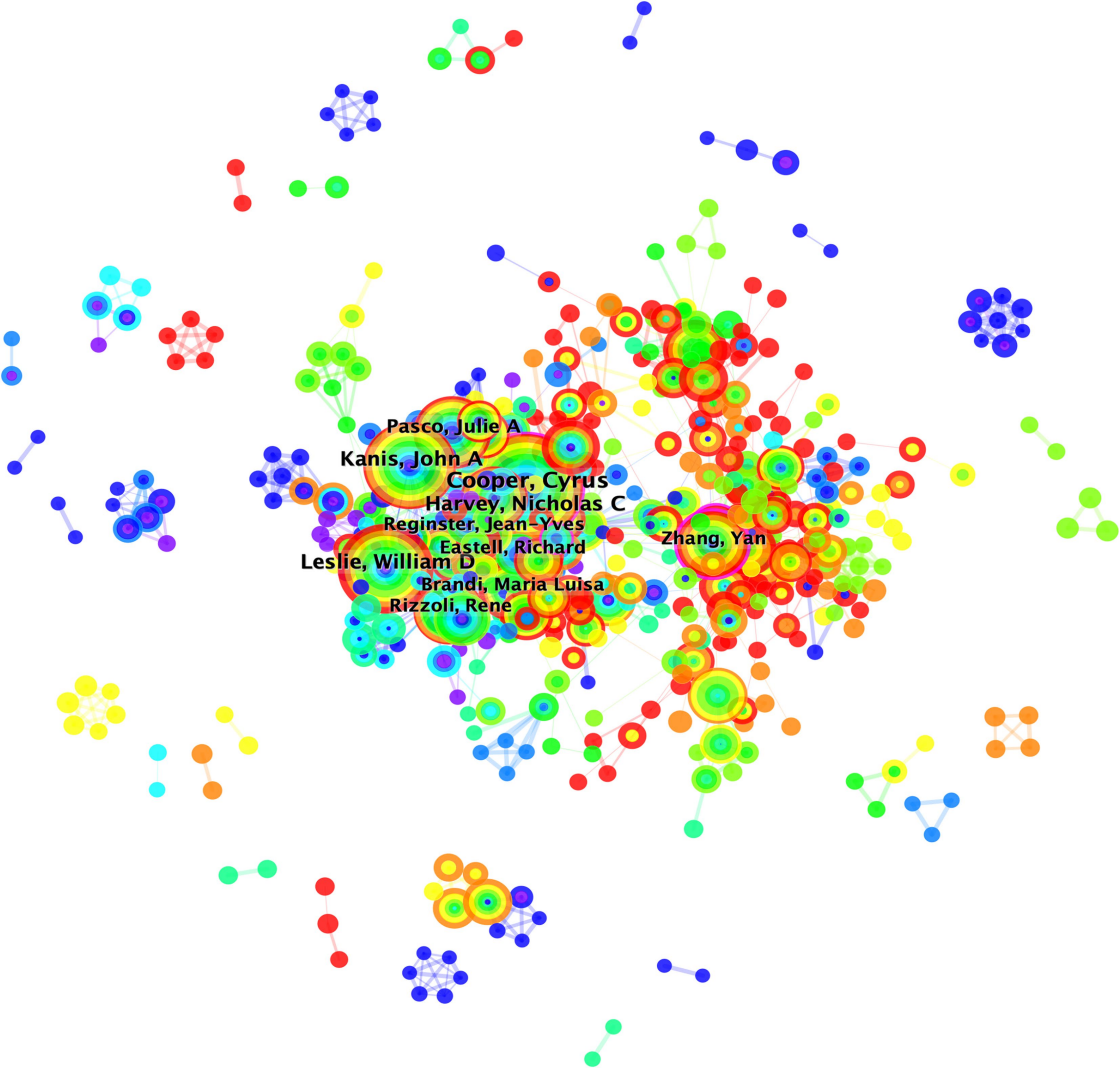
Overlay visualization map of author co-authorship analysis generated by VOSviewer software. The collaboration map of authors reflects the scientific research cooperation between them. The circle/node signifies the authors; size of the circle/node signifies the number of articles. The lines denote the authors’ collaboration strength. The color of the circles transitions from blue to red from the inside out, representing the publication years 2014–2023. This figure is generated using the Citespace software.

An author’s contribution to the scientific literature is quantified by the number of published papers, which reflects their level of engagement in the field (27). The most prolific authors include Cooper Cyrus with 270 publications, followed by Leslie William D (190) and Kanis John A (176; Table 2). Cooper C held the presidency of the International Osteoporosis Foundation and was immersed in aspects such as the pathogenesis (28), diagnosis (29), treatment (30), and prevention (31) of osteoporosis. His involvement extended to several pan-European clinical trials (32, 33) and he contributed significantly to the European guidelines for the diagnosis (34) and treatment of osteoporosis in postmenopausal women, as well as the UK’s clinical guidelines for the prevention and management of osteoporosis (31). WD Leslie’s research primarily focused on fracture risk prediction and diagnosis (35–37). JA Kanis delved deeply into osteoporosis-induced fractures and postmenopausal osteoporosis, participating extensively in the formulation of clinical and treatment guidelines for postmenopausal women in Europe, and engaged in a broad European epidemiological study on osteoporosis (7, 9, 34, 35, 38, 39). The most cited works of these authors were within their respective areas of expertise. The CiteSpace analysis revealed an author collaboration network consisting of 615 nodes and 2,332 links with a density of 0.0124 (Figure 4). Notably, Y. Zhang, E. Michael Lewiecki, L. Zhang, Cyrus Cooper, and J. A. Eisman were at the forefront in terms of centrality measures, with values exceeding 0.1 (Figure 4; Table 2). These findings suggest that osteoporosis research is fragmented and requires greater collaboration among researchers.

**Table 2:** Top 10 active authors in terms of publications and centrality for osteoporosis.

Ranking	Publications	Author	Centrality	Author
1	270	Cooper, Cyrus	0.28	Zhang, Yan
2	190	Leslie, William D	0.27	Lewiecki, E Michael
3	176	Kanis, John A	0.17	Zhang, Lei
4	167	Harvey, Nicholas C	0.13	Cooper, Cyrus
5	126	Pasco, Julie A	0.13	Eisman, John A
6	109	Rizzoli, Rene	0.08	Liu, Qian
7	104	Reginster, Jean-Yves	0.07	Eastell, Richard
8	103	Eastell, Richard	0.07	Deng, Hong-Wen
9	101	Brandi, Maria Luisa	0.07	Yu, Wei
10	100	Zhang, Yan	0.06	Leslie, William D

### 3.3 Overview of research trends and hotspots

#### 3.3.1 Analysis of highly-cited studies

Highly-cited studies possess substantial academic influence within the field (27). Among the top 10 most-cited publications in osteoporosis research, there are 6 original research articles and 4 systematic reviews, with 7 of them being cited over 1,000 times (Table 3) (40–49). Cosman F’s article, titled “Clinician’s Guide to Prevention and Treatment of Osteoporosis,” ranked first with 1990 citations, establishing a comprehensive guideline for the prevention and treatment of osteoporosis (40). Another significant publication is an article by Zhu, Yi titled “The Achilles’ heel of senescent cells: from transcriptome to senolytic drugs,” which was published in 2015 in and ranked second due to its 1,288 citations. This study demonstrated that the combination of dasatinib and quercetin effectively eliminated senescent mouse embryonic fibroblasts, and that periodic drug administration extended healthspan in Ercc1 knock-out mice, thereby delaying age-related symptoms and pathologies, including osteoporosis (41). Ranking third is an update of the guidelines for the diagnosis, evaluation, prevention, and treatment of chronic kidney disease-Mineral and Bone Disorders (CKD-MBD), published by KDIGO in 2017 (42).

**Table 3:** Top 10 most cited publications for osteoporosis.

Ranking	Title	Journal	First author	Publication year	Citations
1	Clinician’s Guide to Prevention and Treatment of Osteoporosis	Osteoporosis International	Cosman, F	2014	1,990
2	The Achilles’ heel of senescent cells: from transcriptome to senolytic drugs	Aging Cell	Zhu, Yi	2015	1,288
3	KDIGO 2017 Clinical Practice Guideline Update for the Diagnosis, Evaluation, Prevention, and Treatment of Chronic Kidney Disease–Mineral and Bone Disorder (CKD-MBD)	Garabed Eknoyan	Kidney Dis Improving Global	2017	1,156
4	Exercise as medicine—evidence for prescribing exercise as therapy in 26 different chronic diseases	Scandinavian Journal of Medicine & Science In Sports	Pedersen, BK	2015	1,148
5	The Recent Prevalence of Osteoporosis and Low Bone Mass in the United States Based on Bone Mineral Density at the Femoral Neck or Lumbar Spine	Journal of Bone and Mineral Research	Wright, NC	2014	1,128
6	Osteoporosis	Lancet	Compston, JE.	2019	1,079
7	Biology of Bone Tissue: Structure, Function, and Factors That Influence Bone Cells	Biomed Research International	Florencio-Silva	2015	1,037
8	Romosozumab Treatment in Postmenopausal Women with Osteoporosis	New England Journal of Medicine	Cosman, F	2016	905
9	Advanced Glycation End Products and Diabetic Complications	Korean Journal of Physiology & Pharmacology	Singh, VP	2014	900
10	Cytokines in Inflammatory Disease	International Journal of Molecular Sciences	Kany, S	2019	859

#### 3.3.2 References co-citation analysis

**Figure 5:**
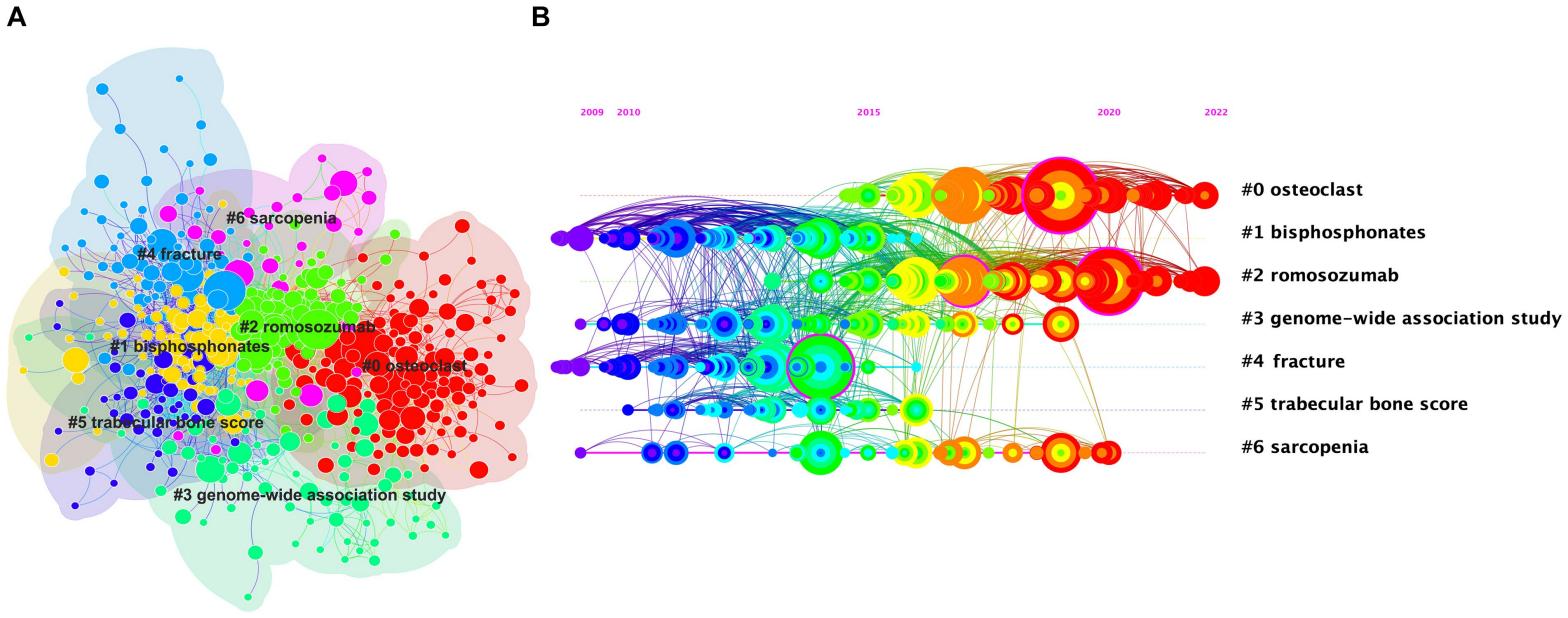
The cluster view map **(A)** and timeline view map **(B)** of reference co-citation analysis were generated by CiteSpace. In the cluster analysis **(A)**, each color represents a cluster, as follows: “0#osteoclasts”—red; “1#bisphosphonates”—yellow; “2#romosozumab”—light green; “3#genome-wide association study”—green; “4#fracture”—blue; “5#trabecular bone score”—purple; “6#sarcopenia”—pink. **(B)** Shows the timeline of reference co-citation analysis, where the color change from purple to red represents the time change from 2009 to 2022. These two figures are generated using the Citespace software.

A reference co-citation analysis is a method used to explore the evolution and boundaries of a particular field (27). The network diagram of co-cited references was visualized and clustered through CiteSpace (Figure 5A). There were seven primary clusters, namely “osteoclast” (#0), “bisphosphonates” (#1), “romosozumab” (#2), “genome-wide association study” (#3), “fracture” (#4), “trabecular bone score” (#5), and “sarcopenia” (#6), each with over 20 nodes. A timeline view that displayed the shift in major clusters over time indicated that the research focus on osteoporosis shifted from primarily “bisphosphonates” (#1), “fracture” (#4), and “trabecular bone score” (#5) towards “genome-wide association studies” (#3) and “sarcopenia” (#6). “Osteoclast” (#0), and “romosozumab” (#2) became research hotspots later but have remained so until the present (Figure 5B).

#### 3.3.3 Keywords co-occurrence analysis

**Figure 6:**
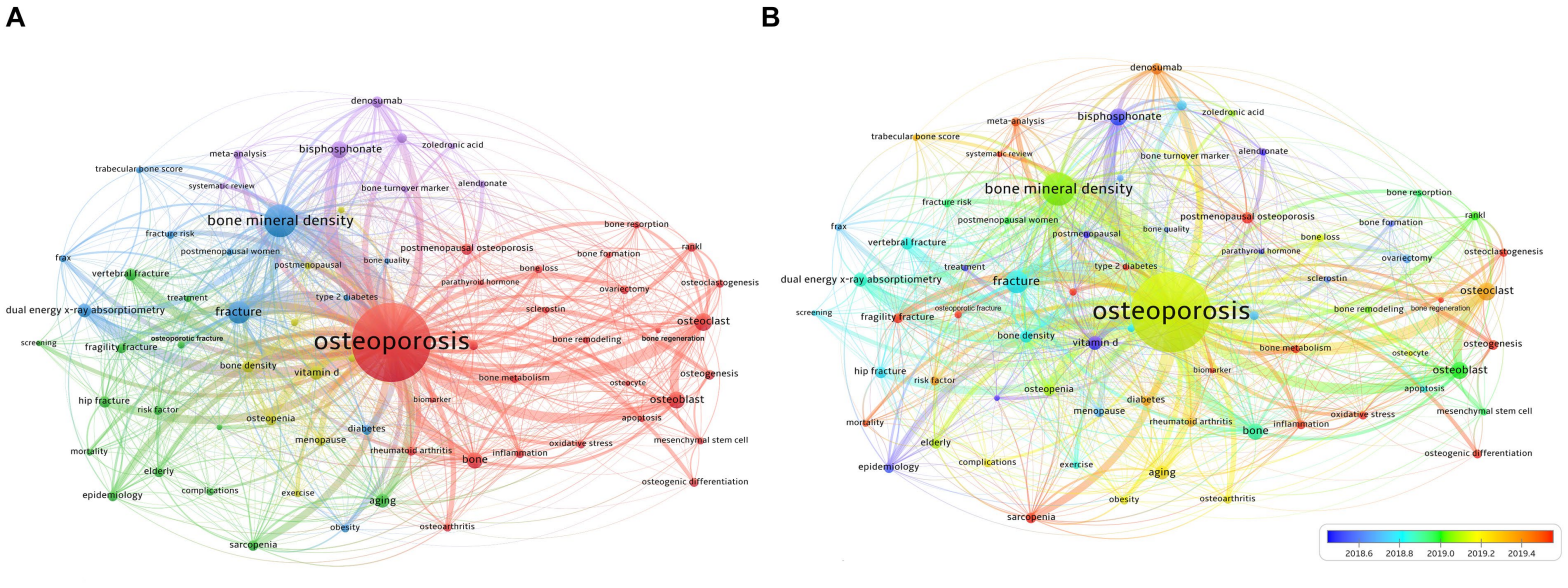
**(A)** Overlay visualization map of keywords co-occurrence analysis. **(B)** The changes in keywords from 2018.6 to 2019.4. **(A)** VOSviewer identified 69 author keywords appearing more than 200 times, categorizing them into 5 principal clusters. These can be summarized from largest to smallest as follows: mechanism (Red), fracture (Green), diagnosis (Blue), prevention (Yellow), and treatment (Purple). **(B)** An overlay visualization depicting the time progression of keywords.

Keyword co-occurrence analysis offers a detailed representation of the thematic scope within the field (27). VOSviewer identified 69 author keywords appearing more than 200 times, categorizing them into 5 principal clusters. These could be summarized from largest to smallest as mechanism (Red), fracture (Green), diagnosis (Blue), prevention (Yellow), and treatment (Purple; Figure 6A). An overlay visualization depicting the time progression of these keywords indicated that “osteogenic differentiation” and “sarcopenia” have been recently discovered and may represent future research hotspots (Figure 6B).

#### 3.3.4 References and keywords burst detection

**Figure 7:**
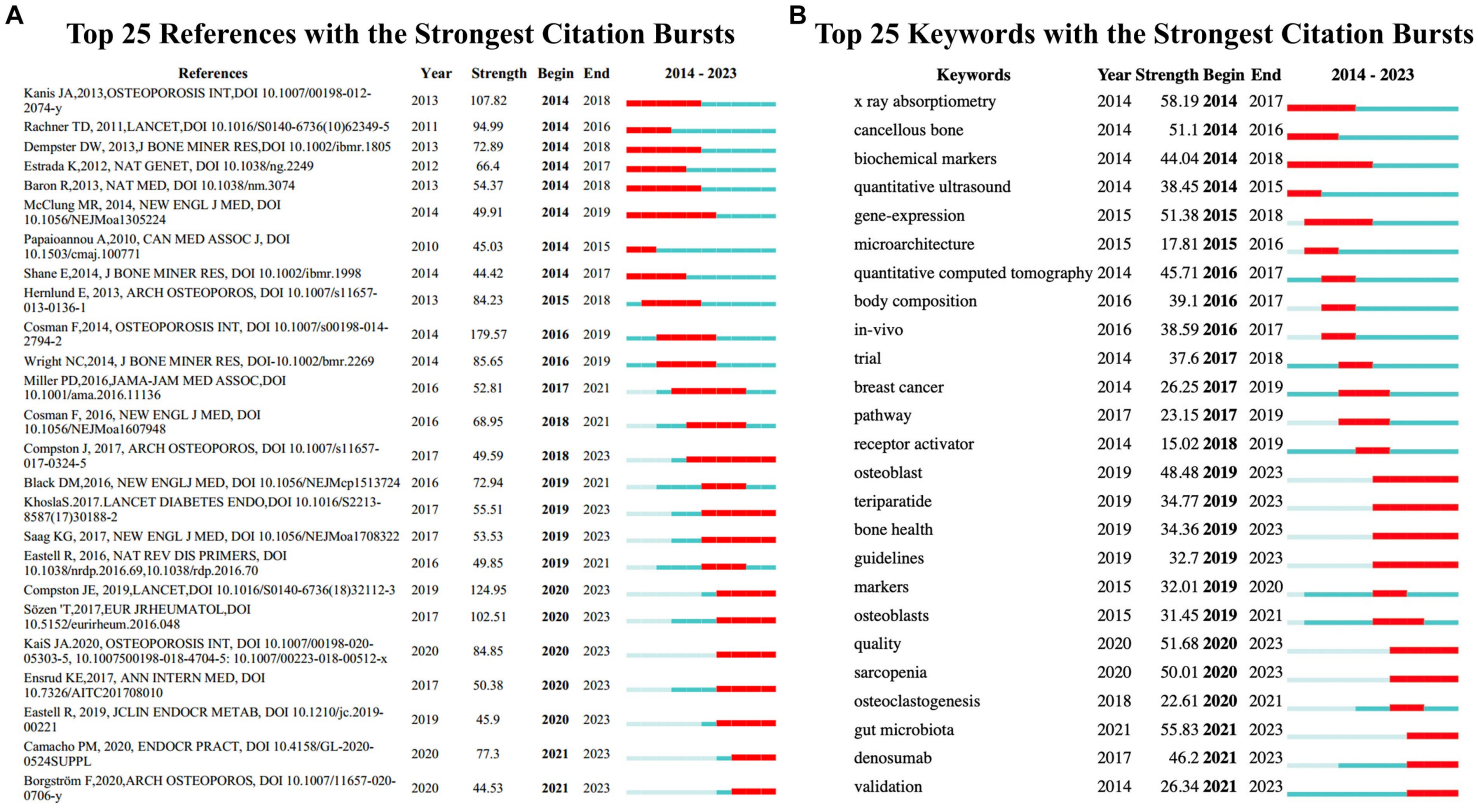
**(A)** References with the strongest citation bursts in publications on osteoporosis research between 2014 and 2023. **(B)** Keywords with the strongest citation bursts in publications on osteoporosis research between 2014 and 2023. The blue lines represent time intervals, while the red segments represent the periods when reference bursts occur. These two figures are generated using the Citespace software.

Burst detection is a method used to recognize sharp increases in the frequency of references or keywords within a specific time period, thereby identifying the most active concepts or topics over time (50). The threshold setting for burst detection was maintained at the default option provided by the software. Red lines indicated the periods when outbreaks of references or keywords occurred (Figure 7). The strongest burst value was associated with a guideline published by Cosman F in 2014, as previously cited (40) (burst value: 179.57). The reference with the second-highest burst value was a review published by Compston JE in 2019 (45). The review highlights that fractures due to osteoporosis are the leading cause of death among older adults. Third on the list is an European guidance for the diagnosis and management of osteoporosis in postmenopausal women (34). The reference with the most persistent burst value was an article by Cosman F titled “Romosozumab Treatment in Postmenopausal Women with Osteoporosis” published in the New England Journal of Medicine in 2016 (47). Additionally, there are 10 references that continue to show high levels of engagement, indicating that these research topics are still receiving attention (1, 4, 31, 34, 45, 51–55). Among these, four original articles were clinical trials on the treatment of osteoporosis, suggesting that the treatment of osteoporosis is currently a highly focused area of research (Figure 7A).

Keywords burst detection identified that x-ray absorptiometry (58.19), gut microbiota (55.83), quality (51.68), gene-expression (51.38), cancellous bone (51.1), sarcopenia (50.01), osteoblast (48.48), denosumab (46.2), quantitative computed tomography (45.71), and biochemical markers (44.04) are the burst keywords with values over 40 (Figure 7B). Five of the above-listed bursting keywords, including osteoblast, quality, sarcopenia, gut microbiota, and denosumab, have shown sustained burst activity up to the present, indicating that these may be related to the new hotspots in osteoporosis research.

## 4 Discussion

### 4.1 Primary findings

This study employed bibliometric methods to analyze 33,928 osteoporosis publications from 2014 to 2023. The total number of publications on osteoporosis has increased over the past decade. The majority of osteoporosis research is concentrated in Western Europe, the United States, and East Asia. Both China and the United States have been prolific in their publication outputs; prior to 2018, the United States led in publication numbers, however, since that year, China has overtaken the United States and maintained the lead. Contributing the most publications was the University of California System, while Amgen achieved the highest centrality. Cooper Cyrus emerged as a leading figure in the field. High-cited studies, co-cited references, and co-occurrence keywords analysis indicated that the past decade’s research predominantly focused on mechanisms, fractures, diagnosis, prevention, and treatment. Burst detection analysis of both references and keywords suggests that current hotspots in osteoporosis research include osteoblasts, sarcopenia, gut microbiota, and denosumab.

### 4.2 Hotspots in osteoporosis researches

The current work explores the research hotspots of osteoporosis through two parts: reference burst analysis and keyword burst analysis. In terms of burst analysis of references, the current research hotspots in the field of osteoporosis are mainly focused on: the clinical diagnosis and treatment of osteoporosis, the fracture risk caused by osteoporosis, and the diagnosis and management of postmenopausal osteoporosis.

Clinical diagnosis and treatment of osteoporosis are key research hotspots in this field. In 2014, Cosman F published clinical guidelines in the field of osteoporosis (37). This guideline, developed by an expert committee of the National Osteoporosis Foundation (NOF) in conjunction with a multispecialty council of medical professionals specializing in bone health convened by NOF, provides clear recommendations on the prevention, risk assessment, diagnosis, and treatment of osteoporosis in postmenopausal women and men aged 50 and older. It includes indications for bone densitometry and establishes fracture risk thresholds for intervention with pharmacological agents. The determination of the absolute risk thresholds above which osteoporosis treatment is advised was based on cost-effectiveness analysis.

The fracture risk caused by osteoporosis is also one of the current research hotspots. In a 2019 review (42), Compston JE highlights that fractures due to osteoporosis are the leading cause of death among older adults. Currently, significant progress has been made in terms of fracture risk assessment; however, many high-risk individuals do not receive adequate assessment and treatment. To address this issue, the recommended approach is to implement integrated care more extensively and establish effective and safe long-term treatment options to consistently reduce the risk of fractures.

The diagnosis and management of osteoporosis in postmenopausal women is also a research hotspot in the field of osteoporosis. The European guidelines for the diagnosis and management of osteoporosis in postmenopausal women (31) have garnered widespread attention in the field of osteoporosis by reviewing several areas, including the role of bone mineral density measurement for diagnosing osteoporosis, assessing fracture risk, general and pharmacological management of osteoporosis, monitoring treatment, fracture risk evaluation, case finding strategies, patient investigations, and the economic aspects of treatment. Cosman F’s study demonstrated that romosozumab is associated with a lower risk of vertebral fractures in postmenopausal women with osteoporosis.

In terms of keyword burst analysis, this study identified several research hotspots in osteoporosis, including osteoblasts, sarcopenia, gut microbiota, and denosumab. Osteoblasts primarily differentiate from mesenchymal progenitor cells located within the inner and outer periosteum as well as the stromal regions of bone marrow. These cells are capable of secreting a diverse array of bioactive substances, which play a crucial role in modulating the processes of bone formation and remodeling (56). Clinically significant disruptions in substrate availability, such as those observed in diabetes mellitus, anorexia nervosa, and aging, can impair osteoblast function, ultimately leading to increased skeletal fragility and the occurrence of osteoporotic fractures (57–59). Recent findings have underscored glycolysis as the principal metabolic pathway that fulfills ATP demands during osteoblast differentiation (60). By altering osteoblast metabolism, it may be possible to effectively enhance both bone quality and mass, potentially offering a therapeutic approach for osteoporosis. In addition to injectable parathyroid hormone (PTH) and its novel formulations, various strategies including PTH-related peptide (PTHrP), calcilytics, beta-adrenergic receptors, the augmentation of Wnt signaling (primarily via sclerostin and Dickkopf-1 neutralization), the regulation of the low-density lipoprotein receptor-related protein (LPR) 5/osteoblast axis, activin, IGF-1, and bone morphogenic proteins (BMPs) have been reviewed for their fundamental rationale and evidence of bone anabolic potential. Sclerostin-neutralizing antibodies, transdermal patches of teriparatide, and PTHrP (1–36) are currently at an advanced stage of research (61).

We have identified sarcopenia as an emerging focal point within osteoporosis research. Sarcopenia is characterized by a progressive and generalized loss of skeletal muscle mass, strength, and/or physical performance (62). The concurrent presence of sarcopenia and osteoporosis has been termed “osteosarcopenia,” which is considered a syndrome (63). While it remains debatable whether individuals afflicted with both sarcopenia and osteoporosis are at a higher risk of falls and fractures compared to those with either condition alone, it is undeniable that the incidence of both conditions increases with an aging population (64, 65). Nevertheless, the mechanisms, epidemiology, and treatment strategies for “osteosarcopenia” remain elusive (11). Clinicians should endeavor to identify and manage sarcopenia in tandem with osteoporosis, particularly in older patient populations.

Increasing evidence suggests that gut microbiota plays a role in bone metabolism, linking bone homeostasis to a healthy microbiome, and indicating that gut dysbiosis could intensify osteoclast activity, exacerbating osteoporosis (66). The relationship between the human gut microbiota, osteoblasts, osteoclasts, and receptor activator of nuclear factor-kappa-B ligand (RANKL) is crucial for modulating osteoclastogenesis and osteoporosis (67, 68). Furthermore, micro-RNA, insulin-like growth factor 1, and immune system mediation are postulated pathways through which the gut microbiome interacts with osteoclastogenesis and bone health in various studies (69–71).

Drug–microbiome interactions have been shown to be integral to therapeutic outcomes and can substantially impact the gut–bone axis (72, 73). Targeting the gut microbiota in osteoporosis therapy offers new therapeutic possibilities and represents a potential opportunity for greater therapeutic control over the natural progression of the disease. Clinicians should give due consideration to the human gut microbiome, taking a holistic approach to patients, especially in treating extra-gastrointestinal conditions such as osteoporosis. A clinician might recommend dietary modification, probiotic-rich foods, or supplementation with probiotics or their metabolites, such as oligosaccharides, carbohydrates, and dietary fiber, aimed at restoring the balance of the gut flora, thereby potentially enhancing bone mineral density by promoting growth and modulating intestinal bacteria composition.

Denosumab, an inhibitor of the receptor activator of nuclear factor kappa-B ligand (RANKL), was approved in 2010 for the treatment of osteoporosis due to its potent antiresorptive activity. This results in clinically significant increases in bone mineral density (BMD) and a decrease in fracture risk at key skeletal sites (74). Over time, concerns about denosumab’s safety and efficacy have been addressed. There is growing clinical consensus and evidence supporting the use of denosumab as an effective treatment for patients at high risk of fractures (75–77). Discontinuation of the drug may lead to an increased risk of multiple vertebral fractures, although there is limited evidence for this effect and how to prevent it (78, 79). Clinicians and patients should be made aware of this potential risk. Based on current data, it is advisable to reassess after 5 years of denosumab therapy. Patients considered at high risk should continue therapy up to 10 years or switch to an alternative treatment. For those at low risk, discontinuing denosumab could be considered after 5 years, but bisphosphonate therapy may be necessary to mitigate or prevent the rebound effect on bone turnover. As the optimal bisphosphonate regimen post-denosumab remains unclear, continued use of denosumab is also an option pending results from ongoing trials (80). Current data suggests that denosumab should not be discontinued without considering an alternative treatment to prevent rapid BMD loss and a potential rebound in vertebral fracture risk. In summary, the available data indicates that denosumab possesses a favorable risk–benefit profile and serves as an adaptable agent for preventing osteoporotic fractures both in the short and long term.

### 4.3 Limitations

The present study has several limitations. Initially, CiteSpace’s development is predicated on the Web of Science (WoS) database; consequently, our choice of the WoSCC database for further inquiry might have precluded the inclusion of pertinent literature from other databases such as Medline, SCOPUS, Cochrane Library, or Google Scholar. Furthermore, the selection was restricted to English-language publications, potentially undervaluing the contributions of non-English literature. Additionally, CiteSpace’s analytical approach is contingent upon citation counts, which, being influenced by various factors, do not exclusively mirror the quality of the articles. Moreover, we did not differentiate between clinical and preclinical papers, aiming instead to provide an encompassing perspective of osteoporosis research. Future analyses focusing on clinical or preclinical studies could offer enhanced insights into practical clinical scenarios or underlying mechanisms. Notwithstanding these limitations, we maintain the belief that the study offers valuable insights into the overarching knowledge structure, evolutionary trends, and key areas of interest in osteoporosis research, thereby guiding the trajectory of subsequent investigations.

## 5 Conclusion

This bibliometric analysis has provided a visualization of the fundamental knowledge frameworks, research trends, and focal points within the realm of osteoporosis. The findings indicate that osteoblast, sarcopenia, gut microbiota, and denosumab represent current hotspots in osteoporosis research, potentially serving as a foundation for future investigative pursuits. Endeavors directed towards elucidating mechanisms, fracture occurrences, diagnostics, and preventive and therapeutic strategies will contribute to a more profound comprehension of osteoporosis.

## Data Availability

The original contributions presented in the study are included in the article/Supplementary material, further inquiries can be directed to the corresponding authors.

## Footnotes

^1^https://bibliometric.com/
